# In vitro metabolism study of α-1,6-glucosylated steviol glycosides

**DOI:** 10.1007/s10068-025-01959-z

**Published:** 2025-07-30

**Authors:** Ye-Lim Park, Mi-Sun Lee, Sung-Hee Park

**Affiliations:** CJ Cheiljedang Research Institute, Suwon, 16495 Republic of Korea

**Keywords:** Stevia, Glucosylated steviol glycosides, Simulated gastric fluid assay, Simulated intestinal fluid assay, Human intestinal microflora, Stevia metabolism

## Abstract

Stevia is a sweetener found in the leaves of the plant *Stevia rebaudiana* Bertoni. It is 50 to 350 times sweeter than sugar and has almost no calories. α-1,6 glucosylated steviol glycosides (GSG) is a novel stevia with a unique chemical structure and improved sensory properties. Considering their potential application in various foods and beverages, we conducted in vitro metabolism study to assess the digestion safety of GSG. In the simulated gastric fluid and simulated intestinal fluid assays, there weren’t significant changes in GSG and steviol glycosides (SG). The anaerobic metabolism study using human fecal homogenates showed complete decomposition of GSG into steviol, similar to common SG such as rebaudioside A, rebaudioside M, and stevia extract, a SG mixture extracted from *Stevia rebaudiana* Bertoni. Overall, the results of the in vitro digestion study show that ingested GSGs can be digested similar to other SG that have established safety for consumption.

**Graphical Abstract:**

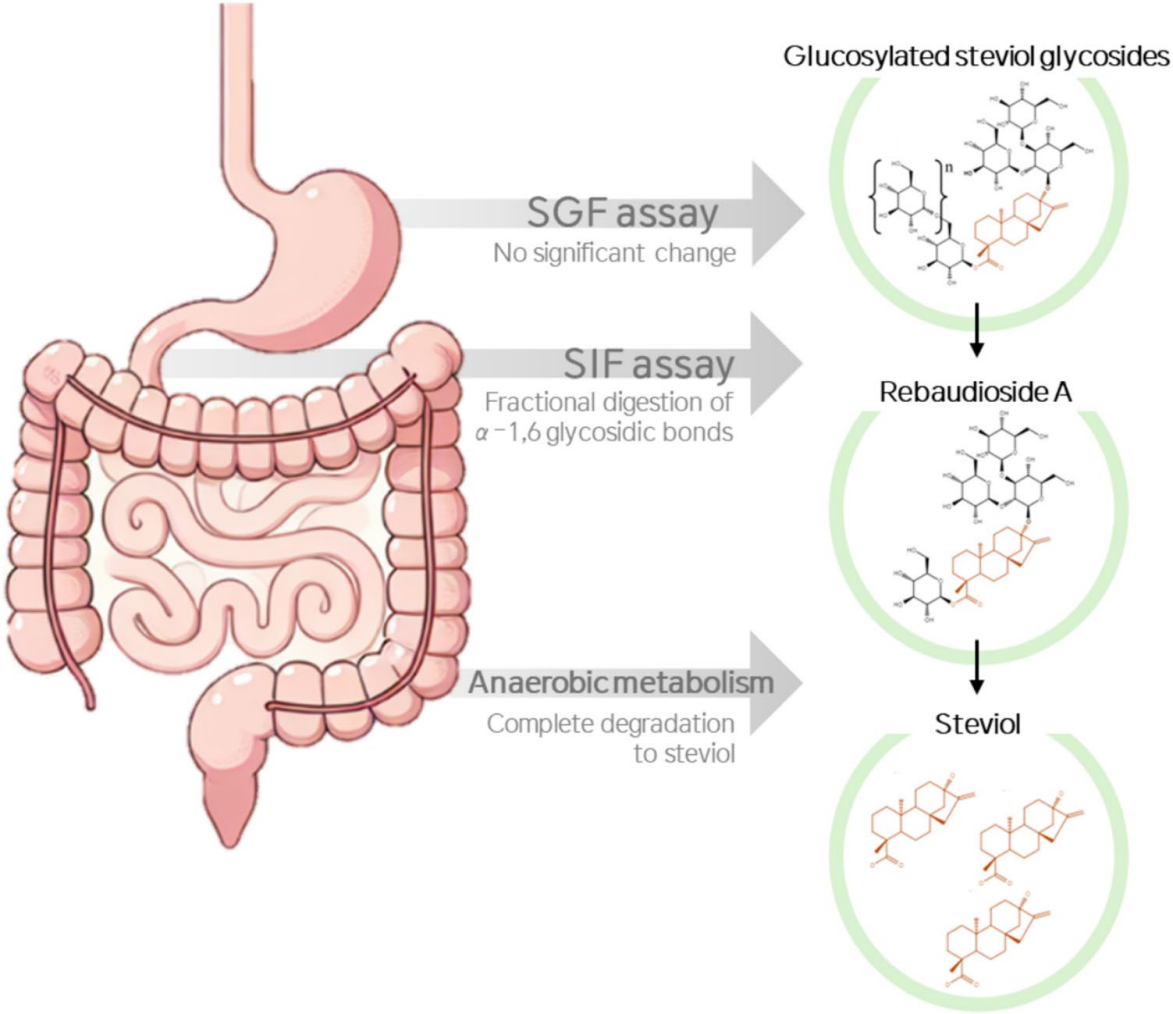

**Supplementary Information:**

The online version contains supplementary material available at 10.1007/s10068-025-01959-z.

## Introduction

Stevia is a sweetener found in the leaves of the plant *Stevia rebaudiana* Bertoni. It is 50 to 350 times sweeter than sugar and has almost no calories, so it is used in various foods and beverages as a sugar alternative (Hossain et al., 2017; Samuel et al., 2018). In particular, stevia has recently been attracting attention as a natural sweetener that can help manage metabolic diseases such as diabetes and obesity (Carrera-Lanestosa et al., 2017; Mohd-Radzman et al., 2013).

Stevioside and rebaudioside (Reb) A are the most abundant steviol glycosides (SGs) in *Stevia rebaudiana* Bertoni leaves and the most commonly used natural alternative sweeteners (Purkayastha and Kwok, 2020; Samuel et al., 2018). However, they are still sensorially distinct from sugar due to their bitterness, astringency, and long-lasting sweetness, which makes their application in foods limited. Reb D and Reb M, which were discovered more recently, are known to have better sensory profiles than Reb A, with less bitterness and more intense sweetness (Celaya et al., 2016; Jung et al., 2021; Zhang et al., 2022). Since Reb D and Reb M exist naturally in very small quantities, their production through enzymatic bioconversion and fermentation methods is being developed. Despite having better sensory profiles than Reb A, the low water solubility and high production costs of Reb M and Reb D still limit their applications (Celaya et al., 2016; Jung et al., 2021; Zhang et al., 2022).

As research on improving the taste of stevia is actively progressing, enzymatically modified stevia (EMS) produced by various methods has recently emerged (Gerwig et al., 2016). EMS is a glucosylated stevia produced through α-glycosylation of SGs with one or more glucose molecules using a glycosyltransferase such as CGTase (Zhang et al., 2023). This conventional EMS has structural characteristics in which glucose molecules are nonspecifically bound to C13 and C19-O-glucose of SGs through α-1,4 linkages (Jung et al., 2021; Kim et al., 2022).

**Fig. 1:**
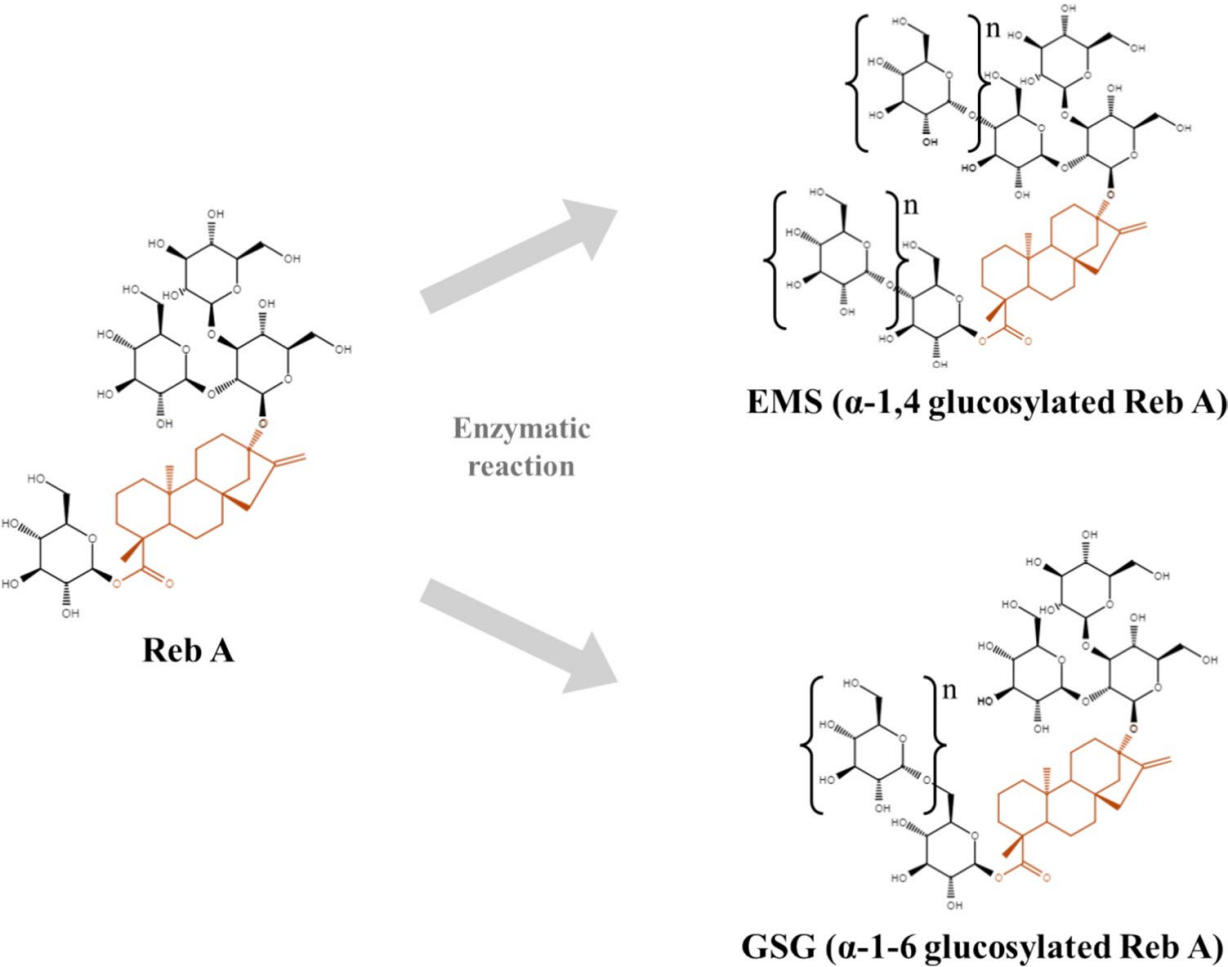
Structures of enzymatically modified stevia (EMS) and glucosylated steviol glycosides (GSG) The structures of two different glucosylated RebA products, EMS and GSG are shown. The steviol backbone is colored orange.

Recently, a structurally distinct form of EMS has been discovered, produced using *Lactobacillus mali, Lactobacillus reuteri, Leuconostoc citreum*, and *Acetobacter capsulatus*, where glucose molecule(s) are attached to the backbone of SGs via α-1,6 linkages (Gerwig et al., 2017; Ko et al., 2016; Te Poele et al., 2018; Yamamoto et al., 1994). Throughout the study, we refer to α-1,6 glycosylated stevia as glucosylated steviol glycosides (GSG). The structural differences between EMS and GSG are illustrated in Fig. 1. Previous studies have shown that GSG has improved sensory profiles compared to Reb A, having less bitter taste, aftertaste, and astringency (Jung et al., 2021; Kim et al., 2022; Muñoz-Labrador et al., 2020).

While the sensory characteristics of GSG has actively been evaluated, no research has been conducted to prove its digestion-related safety (Kim et al., 2022). The metabolic mechanisms of SGs produced by various methods and the effects of their breakdown products on the human body have been extensively studied (Koyama et al., 2003a, 2003b; Nikiforov et al., 2013). It is known that the metabolism of GSG is sufficiently similar to already approved SGs to support their safety as food additives (EFSA FAF Panel et al., 2022). Previous studies have confirmed the digestion of EMS to SGs, which are degraded to steviol by gut microflora (Koyama et al., 2003a), metabolized to glucuronide in the liver, and finally excreted in the urine (Geuns et al., 2006; McQuate, 2011). GSG has the same backbone structure as other stevia types, with a few extra glucose molecules attached to Reb A, and is expected to be metabolized similarly. Nevertheless, the unique structure of GSG having α-1,6 glucosylation may require distinct safety evaluations.

In this study, we evaluated the digestion safety of GSG (α-1,6 glycosylated stevia) through in vitro metabolism experiments, using methods similar to those applied in previous digestion studies of SGs. We conducted in vitro simulated gastric fluid (SGF) assay, simulated intestinal fluid (SIF) assay, and anaerobic metabolism study using human fecal homogenates (HFHs) to confirm that the metabolic fate of GSG is similar to that of the well-known types of stevia (Reb A, Reb M, and stevia mixture). Through these studies, we demonstrated the complete degradation of GSG into steviol, thereby supporting the safety of GSG consumption.

## Materials & methods

### Chemicals & human stool materials

Pepsin, NaCl, HCl, Na_2_CO_3_, KH_2_PO_4_, bovine serum albumin (BSA), pancreatin, starch, and dimethyl sulfoxide (DMSO) were purchased from Sigma-Aldrich (USA). Methanol was purchased from Fisher Scientific (USA). GSG was obtained from CJ CheilJedang Research Institute. Reb A, Reb M, and stevia extract—a mixture of SGs extracted from *Stevia rebaudiana* Bertoni—were purchased from Haigen Bio-Tech (China). The detailed information of the stevia extract is provided in Supplementary data 1. Gels and reagents for sodium dodecyl sulfate–polyacrylamide gel electrophoresis (SDS-PAGE) were purchased from Bio-Rad Laboratories, Inc. (USA). The D-Glucose assay kit for the glucose oxidase/peroxidase (GOPOD) method was purchased from Megazyme (Ireland).

The human fecal test was conducted at Biopharmaceutical Research Inc. (BRI; Vancouver, Canada), and for the study, deionized water (DW; BRI in house), methanol (Fisher Scientific), DMSO (Sigma), potassium phosphate buffer saline (PBS) pH 7.4 (Sigma), brain heart infusion (BHI) broth (Oxyrase Inc.), Oxyrase® for Broth (Oxyrase Inc.), and steviol hydrate (Sigma) were used. The stool materials from six healthy male and female adult donors were collected and provided by BioIVT (NY, USA). To conduct experiments using fecal samples with a stable and healthy gut microbiota, the following study inclusion and exclusion criteria were established. 1. Healthy adults aged between 18 and 65 years old with body mass index below 30 kg/m^2^. 2. No history of gastrointestinal diseases. 3. No history of diabetes or cardiovascular disease or related medications. 4. Absence of oral ingestion of prebiotic, fiber supplements, or probiotic supplements at least 14 days prior to stool collection. 5. Absence of oral ingestion of stevia, lactulose, sucralose sweeteners, laxatives, and antimicrobial drugs at least 14 days prior to stool collection. 6. Not pregnant or lactating. The human subject collection methods were approved by the Institutional Review Board (IRB) WCG with Federal Wide Assurance (IRB No.: FWA00015953).

### SGF assay

The SGF solution was prepared with 3.2 mg/mL pepsin in 0.03 M NaCl (pH 1.2), and the aliquots of 200 μL SGF solution were preincubated at 37 ℃ for 2 min in a water bath. Additionally, 5 mg/mL GSG, Reb A, Reb M, stevia extract, and BSA samples were prepared in PBS buffer and 10 μL of each sample solution was added to preheated SGF solution. At 0, 1, 5, 10, 30, 60, and 120 min after the reaction, 75 μL of 0.2 M Na_2_CO_3_ was added to each sample for inactivation of pepsin. After filtration, these samples were analyzed using high-performance liquid chromatography (HPLC), where the remaining GSG, Reb A, Reb M, and stevia extract were measured. To measure the stevia extract’s amount, the sum of the peak areas of Reb A and stevioside was calculated. The separation of the analytes was performed on the Capcell-pak C18 MG-II column (Agilent, Santa Clara, CA, USA). Furthermore, an isocratic elution with 30% acetonitrile in water at a flow rate of 1 mL/min was applied at 40 ℃. The analytes were detected under UV absorbance at 210 nm. SDS-PAGE was utilized to confirm pepsin activity in the SGF solution by analyzing the residual BSA following incubation. BSA samples were treated with SDS sample buffer solution and heated for 5 min at 95 ℃. The samples were loaded on a 12% acrylamide gel (Bio-Rad) and separated at 120 V for 35 min. The gel was stained with Instant Blue Coomassie solution (Expedeon) and analyzed using Gel DocTM EZ Imager (Bio-Rad, USA).

### SIF assay

The SIF solution was prepared with 10 mg/mL pancreatin in 0.05 M KH_2_PO_4_ (pH 7.5), and the aliquots of 64 μL SIF solution were preincubated at 37 ℃ for 2 min in a water bath. Additionally, 5 mg/mL GSG, Reb A, Reb M, stevia extract, and starch samples were prepared in PBS buffer, and 10 μL of the sample solution was added to the preheated SIF solution. At 0, 1, 5, 10, 30, 60, and 120 min after the reaction, samples were inactivated by heating at 95 ℃ for 10 min in a heating block. The samples were analyzed using HPLC with the same method described for the SGF assay. For the detection of glucose in stevia and starch digestions, the GOPOD method was used. The amount of glucose hydrolyzed from stevia and starch was determined spectrophotometrically at 510 nm after 20 min of incubation at 50 °C with the GOPOD reagent (Megazyme).

### In vitro* anaerobic metabolism in pooled HFHs*

The total study outline of in vitro anaerobic metabolism of SGs is provided in Supplementary data 2. Additionally, 10–20 g wet weight of fecal material from each donor was immediately collected in a container prefilled with 2% Oxyrase in 50 mL 0.01 M isotonic phosphate buffer (pH 7.4). Fecal materials from six subjects of the same gender were pooled (n = 6) to afford a total of one pooled male adult fecal homogenate and one pooled female adult homogenate. Two pooled fecal homogenates were homogenized by shaking and mixing with a stirrer. Under anaerobic conditions, the supernatant of each pooled fecal homogenate was transferred to another container and diluted with the BHI broth to provide a 50X diluted pooled HFH. The 50X diluted pooled HFH was preincubated at 37 °C overnight for next-day use. Additionally, GSG, Reb A, Reb M, stevia extract, and steviol were independently dissolved in DMSO to provide 50X stock solutions at a concentration of 10 mg/mL.

For the main study, GSG as a test article and Reb A, Reb M, and stevia extract as positive controls were incubated independently at a target concentration of 200 μg/mL in each sample of male and female pooled HFHs under anaerobic conditions at 37 °C in triplicate at each of 0, 4, 8, 12, 24, 48, 72, and 96 h. Incubation of the samples was performed in 2 mL polypropylene Lok-capped vials (Eppendorf). Reb A, Reb M, and stevia extract were used as comparison groups in the same test. Furthermore, 10 μL of GSG, Reb A, Reb M, and stevia extract stock solutions were accurately dispensed into each sample vial, followed by the addition of the 0.5 mL preincubated fecal homogenate to each vial, performed inside an anaerobic nitrogen hood pre-purged with high-purity nitrogen to ensure < 1% oxygen level. The samples were capped, vortex mixed, and incubated at 37 °C ± 2 °C in a calibrated environmental chamber. At the end of each incubation time point, 1 mL of methanol as a stop solvent was added to each sample with vortex mixing to stop the reaction. The samples at the 0-h time point were pretreated with the methanol stop solvent prior to the spiking of fecal homogenate and test materials.

The negative control test involved inoculating GSG, Reb A, Reb M, stevia extract, and steviol stock solutions into BHI broth without fecal homogenate, maintaining the same incubation conditions as the main study to verify the stability of the test materials throughout the incubation period. In addition, the blank pooled HFH samples were prepared to prove the absence of an interference peak at the retention time of steviol. All tests were performed in triplicate, and all samples were stored at − 70 °C before proceeding with liquid chromatography–mass spectrometry (LC/MS).

### LC/MS analysis

For LC/MS analysis, isosteviol and siamenoside I (Sigma), which are structurally similar to steviol and Reb A, were used as LC/MS assay internal standards. Test samples were equilibrated to room temperature, followed by sonication for 5 min, vortex mixing for 1 min, and spinning at 12,000 rpm for 5 min at room temperature. An aliquot of the internal standard solution was spiked into each sample, except for the blanks. Samples were further diluted with 50% methanol in DW. The resulting concentration of the internal standard in each diluted sample was 100 ng/mL of isosteviol and 400 ng/mL of siamenoside I. Following vortex mixing and centrifugation to remove protein precipitates, the supernatant was transferred to LC vials for LC/MS analysis.

Furthermore, samples were separated on the Luna C18 column (30 X 2 mm, Phenomenex, USA) by a gradient solvent system consisting of acetonitrile and HPLC water with a 0.3 mL/min flow rate and 10 μL injection volume using ESI negative SIR MS mode. LC/MS system was carried out with an Agilent Model 1100 HPLC system coupled with a Waters Micromass Quattro^ⓡ^-Micro triple quadrupole mass spectrometer and controlled by Waters Micromass Masslynx^ⓡ^ software Version 4.0. Supplementary data 3 shows the analyzed m/z of each analyte (GSG, Reb A, Reb M, stevia extract, isosteviol, and siamenoside I) using LC/MS.

## Results and discussion

### SGF assay

**Fig. 2:**
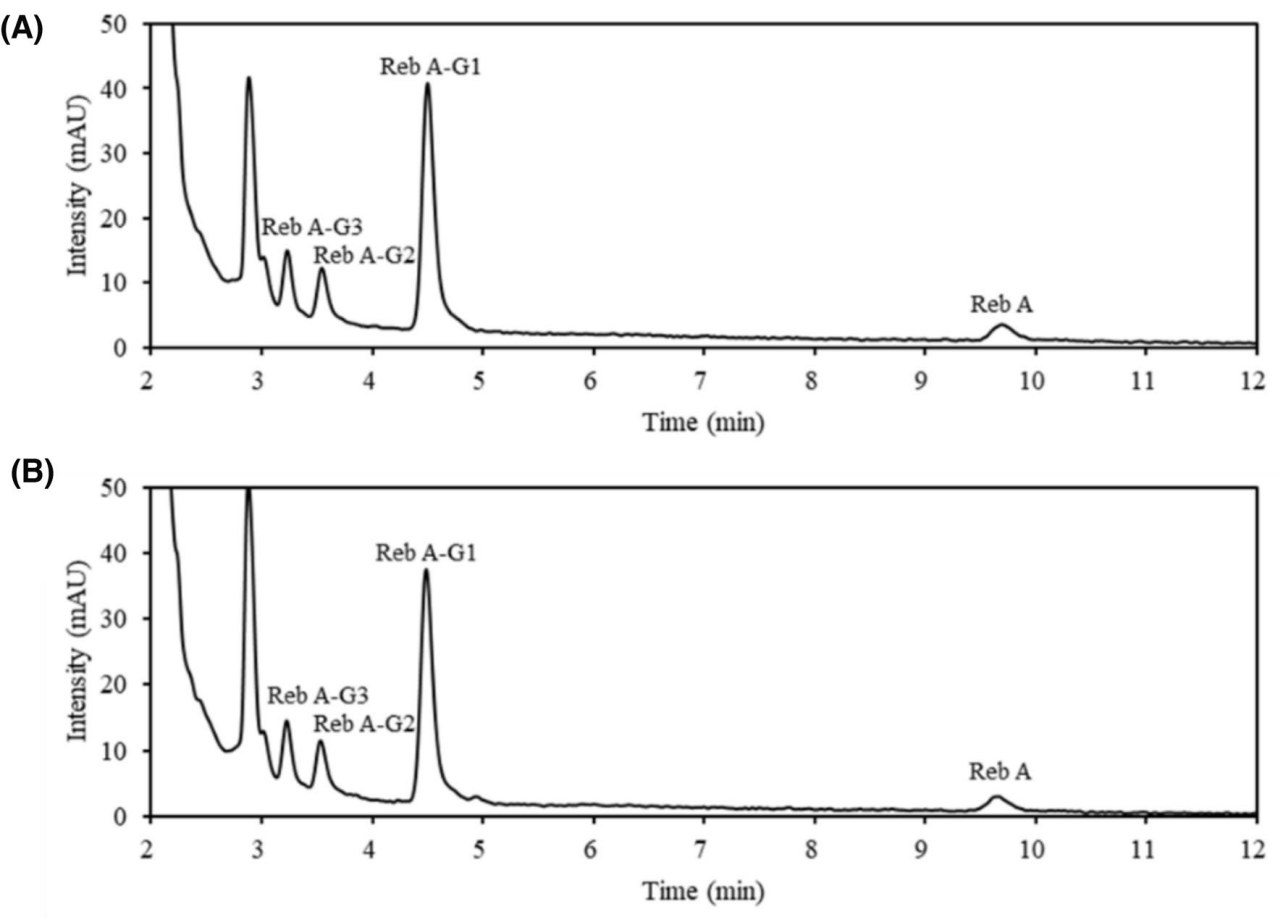
High-performance liquid chromatography (HPLC) results of glucosylated steviol glycosides (GSGs) digested by simulated gastric fluid (SGF) assay solution. *Reb A-G1* Monoglycosylated-Reb A, *Reb A-G* Diglycosylated-Reb A and *Reb A-G3* Triglycosylated-Reb A. **A** HPLC chromatogram of GSG at 0 h of incubation and **B** after 4 h of incubation with SGF solution

To evaluate the human intestinal digestion stability of GSG, in vitro SGF and SIF assays were primarily performed. HPLC results of the SGF assay showed no changes in GSGs until 4 h of incubation (Fig. 2). The same reaction was performed on the general SGs, such as Reb A, Reb M, and stevia extract. Similarly, there were no changes until 4 h of incubation (Supplementary data 4). Therefore, GSG is stable as general SGs under conditions simulating the human stomach. Previous studies have shown that SGs and EMS become less stable at lower pH levels and higher temperatures (Chaturvedula et al., 2011a; 2011b; Glenn Sipes et al., 2016; Prakash et al., 2012). However, the above data indicate that GSG pass through the stomach with minimal alterations, given their typical residence time of less than 2 h (Leiper, 2015). The complete degradation of BSA within 1 h confirmed the efficiency of the gastric juice enzyme used, suggesting that GSG is genuinely stable under the tested conditions (Supplementary data 5).

### SIF assay

**Fig. 3:**
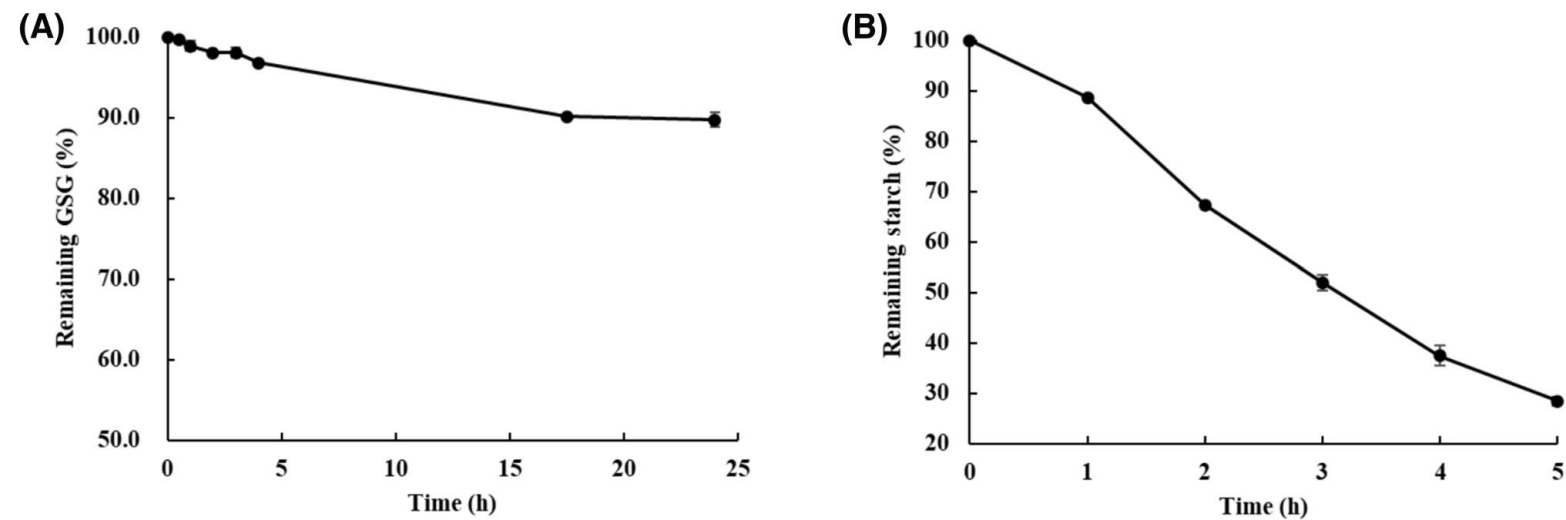
Plots of glucosylated steviol glycoside (GSG) and starch degradation in simulated intestinal fluid (SIF) assay. **A** Result of GSG degradation analyzed using HPLC after 24 h of incubation and **B** result of starch degradation analyzed by GOPOD method after 5 h of incubation

To study GSG degradation in the duodenum, the SIF assay was performed and analyzed using HPLC after GSG samples were incubated with the SIF solution for 0–24 h. HPLC analysis in the SIF assay showed no significant changes in GSG content for up to 1 h of incubation. The SIF solution did not significantly degrade GSG (Fig. 3 (A)). Additionally, the glucose measurement test was performed using the GOPOD method to determine released glucose molecules from glucosylated Reb A; however, the glucose content was below the detection limit.

We have tested decomposition of starch as a measure of the α-amylase activity. The GOPOD assay showed that 71.6% ± 0.8% (w/w) glucose from starch was released within 5 h (Fig. 3 (B)) The assay conducted in this study utilized an enzyme concentration significantly exceeding that found in the intestinal environment at any given time, aiming to demonstrate potential GSG degradation by digestive enzymes (Takagi et al., 2003; Yagami et al., 2000). Our finding indicated that a small amount of GSGs is converted to RebA in the presence of excess pancreatin, suggesting that the majority, if not all, of GSGs likely passes through the intestinal phase undecomposed. Based on the results of the SGF and SIF assays, considering the amount of enzyme used and the residence time in the stomach and intestine (Sensoy, 2021), it is anticipated that the GSG will not undergo significant degradation upon ingestion.

### Anaerobic metabolism of GSG in pooled HFHs

It is known that GSG and SGs are broken down into steviol, the backbone of SGs, by the intestinal microflora and then transported to the liver to be metabolized into glucuronide (Koyama et al., 2003a). The glucuronide is then secreted through urine; therefore, the stevia consumption is regarded as safe (Geuns et al., 2006; Younes et al., 2022). Thus, we assessed the degradation of GSGs by quantifying the steviol produced as a fecal test product.

**Fig. 4:**
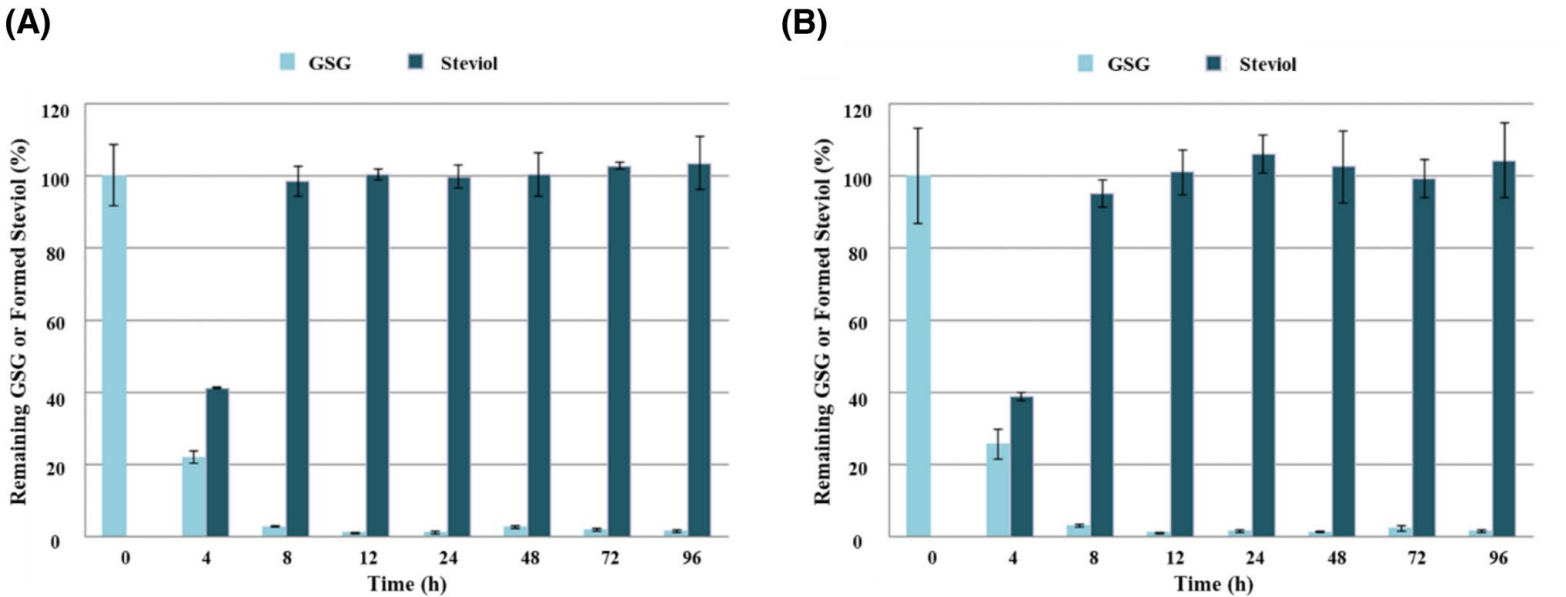
Plots of % GSG remaining and % steviol formed from GSG at each incubation time points over 96 h in fecal homogenate. **A** Result of male fecal homogenate and **B** result of female fecal homogenate

Figure 4 shows the corresponding plots of % GSG and total steviol molar equivalent formed from GSG in pooled HFHs. This data indicated that about 74–78% deglycosylation of the parent GSG occurred within 4 h in both adult male and female fecal homogenates. Within 12 h of incubation, almost complete deglycosylation of GSG to steviol was achieved. However, the total molar equivalent of steviol formed was only observed at a mean of 41% in males and 39% in females at the 4 h time point. This relatively low formation of total molar equivalent of steviol observed at 4 h suggested the stepwise occurrence of deglycosylation involving partially deglycosylated intermediates such as stevioside and Reb A. When near complete deglycosylation to steviol was achieved at the 12 h time point, the total molar equivalent of steviol was observed at a mean of 100% ± 2% in males and 101% ± 6% in females (Fig. 4). Therefore, deglycosylation of GSG was occurring in a comparable manner in the pooled fecal homogenates from adult male and female donors.

**Fig. 5:**
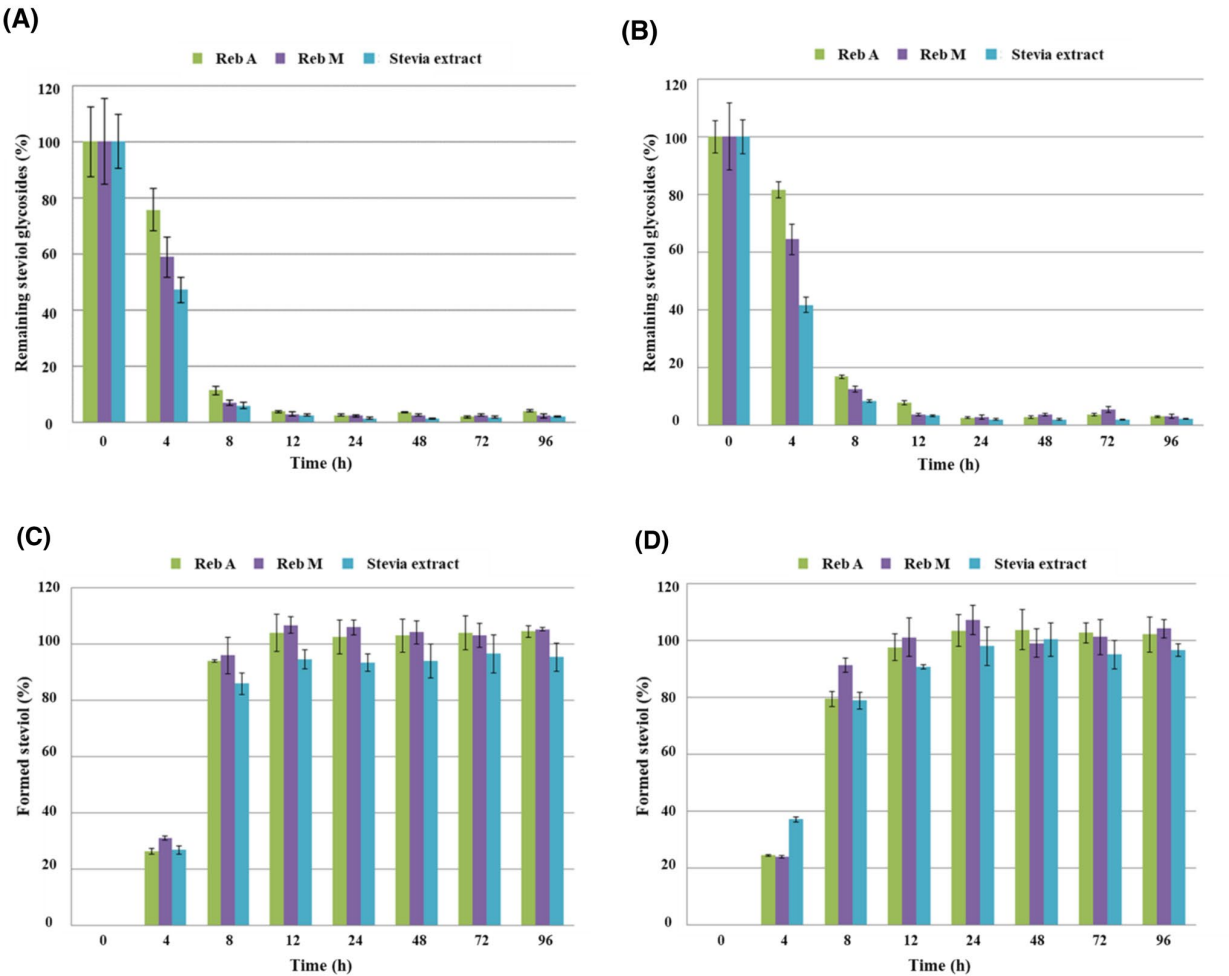
Plot of % remaining positive controls (Reb A, Reb M, and stevia extract) and their formed steviol levels at each incubation time points over 96 h in adult fecal homogenates **A** Result of remaining Reb A, Reb M, and stevia extract in male fecal homogenate, **B** result of remaining Reb A, Reb M, and stevia extract in female fecal homogenate, **C** result of formed steviol in male fecal homogenate, and **D** result of formed steviol in female fecal homogenate

The results of the positive control group Reb A, Reb M, and stevia extract also confirmed that most SGs were degraded in the fecal homogenate of males and females within 12 h of incubation time (Fig. 5 (A) and (B)). It was also confirmed that as SGs decomposed, the amount of steviol formed increased accordingly (Fig. 5 (C) and (D)).

### Negative control test and blank of HFHs

A negative control test was performed to confirm the stability of SGs under the same incubation conditions. Reb A, Reb M, stevia extract, and GSG as negative controls in BHI broth without fecal homogenates at a total nominal concentration of 0.2 mg/mL were also conducted under anaerobic conditions at a nominal 37 °C in triplicate at each of 0, 4, 8, 12, 24, 48, 72, and 96 h. The data of negative controls confirmed that the amount of Reb A, Reb M, stevia extract, and GSG remained unchanged over 96 h of incubation in BHI broth without HFHs, and no steviol was detected at each of the incubation timepoints (Supplementary data 6). Additionally, to confirm the stability of steviol in HFHs, it was incubated in pooled HFHs at a total nominal concentration of 0.2 mg/mL under the same incubation conditions for 96 h. It was confirmed that steviol was very stable, with an average content of 100.4% ± 2.3% of total remaining steviol (Supplementary data 7). Each male and female fecal homogenate was analyzed using LC/MS to check for any interference with the retention time of steviol, and it was confirmed that no peak was observed at the same retention time (Supplementary data 8). These control studies confirmed that the intestinal microflora degraded GSG to steviol, not spontaneously, under the experimental conditions.

Given the potential use of GSG in a wide range of foods and beverages, we sought to ensure its safety for consumption through indirect metabolism studies. As shown by the HFH test results, GSG passing through the small intestine is completely digested to steviol by the intestinal microflora. Steviol is expected to be metabolized in the liver to glucuronide and excreted in the urine, as previously shown in metabolism studies of various SGs (Koyama et al., 2003a; Purkayastha and Kwok, 2020; Purkayastha et al., 2016, 2014). The findings of this study can support the safety of this novel stevia, as GSG share a similar metabolic pathway with other SGs that have established safety for consumption.

## Supplementary Information

Below is the link to the electronic supplementary material.

Supplementary file1 (PDF 317 KB)
