# Supplementary material for: In vitro metabolism study of α-1,6-glucosylated steviol glycosides
Source: Food Sci Biotechnol. 2025 Jul 30;34(15):3729–37. doi: 10.1007/s10068-025-01959-z (PMC12528587; doi:10.1007/s10068-025-01959-z)

Supplementary data 1. HPLC chromatogram and the composition of stevia extract : The product name is RA40SG95 and purchased from Haigen Bio-Tech.

The HPLC chromatogram shows the separation of the stevia extract contents. The composition of the each steviol glycosides contents are shown in the table below.

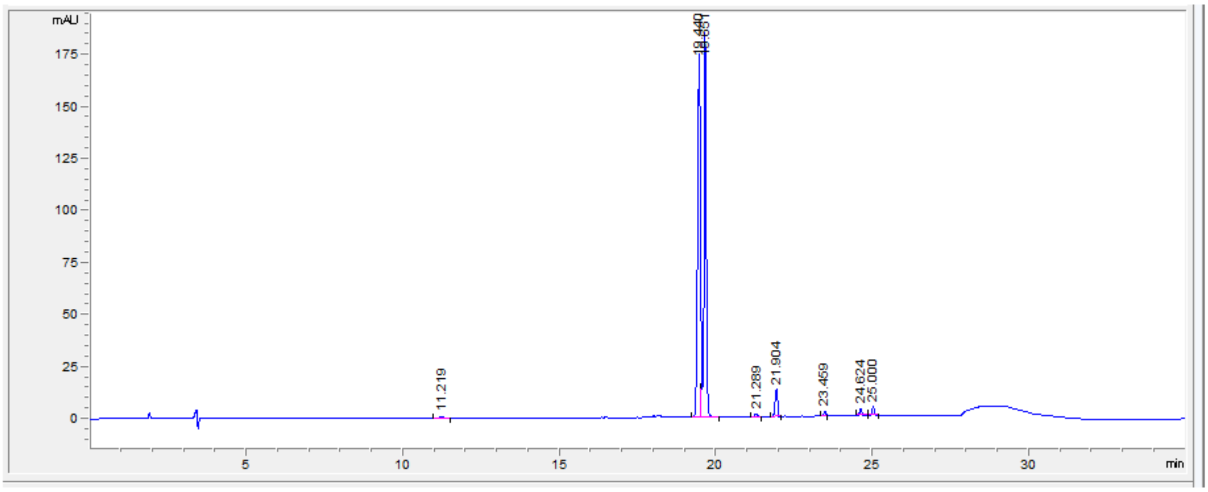

No.	Steviol glycosides	Retention time	Height	Width	Area	Con. %
1	Reb D	11.219	1	0.1432	9.7	0.40
2	Reb A	19.44	175	0.095	1065.7	45.41
3	Stevioside	19.651	184.6	0.0957	1133.9	48.32
4	Reb F	21.289	1.5	0.1088	10.7	0.46
5	Reb C	21.904	13.1	0.091	77.7	3.31
6	Dulcoside A	23.459	2.4	0.0722	11.2	0.48
7	Reb B	24.624	3.2	0.0755	15.7	0.67
8	Steviolbioside	25.000	4.2	0.082	22.2	0.95

Supplementary data 2. Study outline of *In Vitro* Anaerobic Metabolism test

<table>
<tr><td>Study Materials</td><td>Sample Conc. (mg/mL)</td><td>LC/MS Assay for Parent-Disappearance and steviol Metabolite Formation</td><td>Incubation time point</td></tr>
<tr><td>Test Material (GSG)</td><td>0.2</td><td rowspan="3">Analysis of disappearance of steviol glycosides & molar equivalent formation of Steviol</td><td rowspan="3">0, 4, 8, 12, 24, 48, 72 and 96 hr</td></tr>
<tr><td>Positive control (Reb A, Reb M and Stevia extract)</td><td>0.2</td></tr>
<tr><td>Negative Control (Reb A, Reb M, Stevia extract, GSG, steviol)</td><td>0.2</td></tr>
<tr><td>Blank Control (HFH)</td><td>N/A</td><td>Analysis of HFH without steviol</td><td>0 hr</td></tr>
</table>

Supplementary data 3. Analyzed m/z of GSG, Reb A, Reb M, Stevia extract, Isosteviol and Siamenoside I by LC/MS analysis

Analyte	MW	m/z monitored
GSG (main peak)	1128 (RebA-G1 as marker)	$[M-H-2glc]^-$<br>$[M-H]^-$
Reb A	967	$[M-H-2glc]^-$<br>$[M-H-glc]^-$<br>$[M-H]^-$
Reb M	1291	$[M-H]^-$<br>$[M-2H]^{-2}$
Stevia extract (main peak)	967 (Reb A as marker)	$[M-H-2glc]^-$<br>$[M-H-glc]^-$<br>$[M-H]^-$
Isosteviol	318	$[M-H]^-$
Siamenoside I	1125	$[M-H]^-$

Supplementary data 4. Mean % of remaining steviol glycosides after incubation in SGF and SIF assay

	% Parent remaining					
Assay	SGF assay			SIF assay		
Reaction time	Reb A	RebM	Stevia extract	Reb A	RebM	Stevia extract
0h	100.0	100.0	100.0	100.0	100.0	100.0
1h	97.8	105.6	103.8	101.9	104.0	98.5
2h	99.3	105.8	105.0	100.1	104.5	105.0
3h	93.4	104.2	99.5	97.2	103.8	100.1
4h	103.9	101.2	100.0	103.3	101.7	103.1

Supplementary data 5. SDS PAGE result of BSA digested by SGF assay solution Lane 1: SDS PAGE Ladder, Lane 2: BSA sample (0.175mg/ml, same as BSA concentration in SGF digestion mixture), Lane 3: SGF assay solution (pepsin included), Lane 4: Mixture of BSA and SGF solution after 0h digestion, Lane 5: 1h digestion, Lane 6: 2h digestion, Lane 7: 3h digestion, Lane 8: 4h digestion

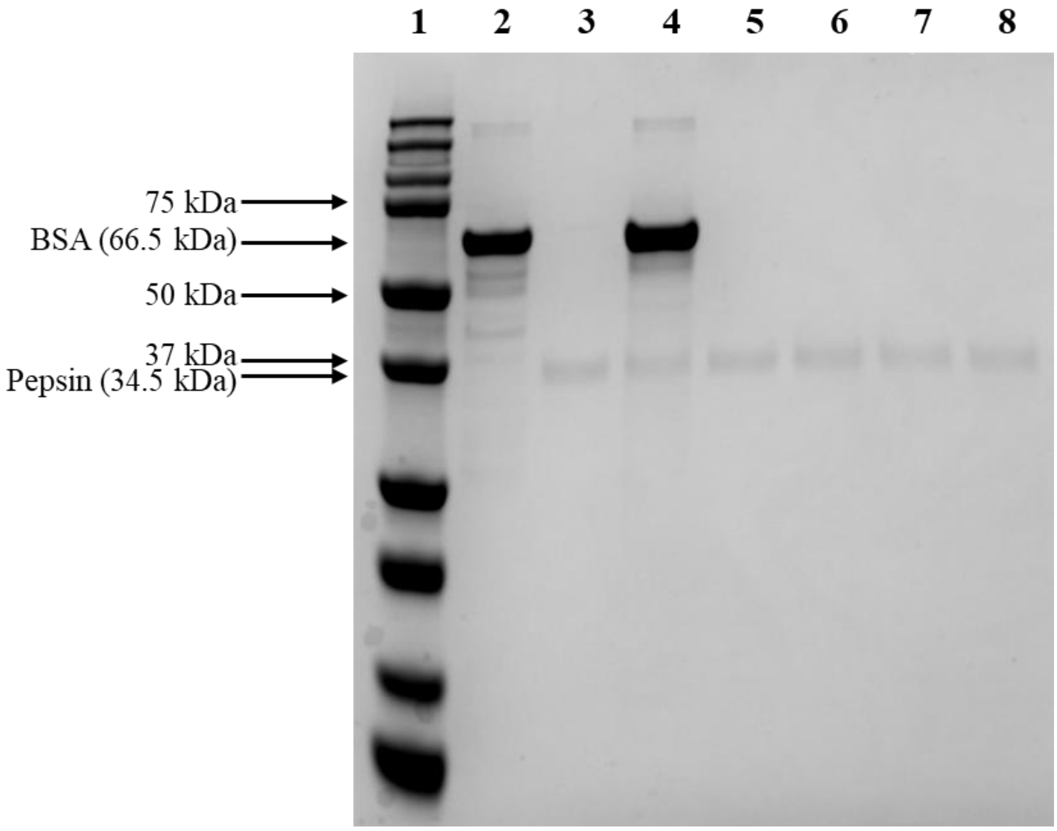

Supplementary data 6. Results of negative control test

Summary of mean % of steviol formed and parent compounds remaining (Reb A, Reb M, stevia extract and GSG) detection at each incubation time point up to 96 hours in BHI broth without HFH.

Timepoint (hr)	% Steviol Metabolite Formed				% Parent Remaining			
	Reb A (%)	Reb M (%)	Stevia extract (%)	GSG (%)	Reb A (%)	Reb M (%)	Stevia extract (%)	GSG (%)
0	No Steviol Peak Detected	No Steviol Peak Detected	No Steviol Peak Detected	No Steviol Peak Detected	100.0	100.0	100.0	100.0
4					103.0	107.1	98.0	103.1
8					102.3	105.9	100.4	103.2
12					102.4	102.5	96.8	100.4
24					97.5	101.8	95.8	105.0
48					100.1	102.5	92.5	100.4
72					100.2	98.3	92.7	102.2
96					99.9	104.3	93.3	105.5

Supplementary data 7. Summary of mean % steviol remaining as a stability control at each incubation time point up to 96 hours in pooled HFH (mixture of adult male and adult female pooled fecal homogenate)

Timepoint(hr.)	% Steviol Remaining in Pooled HFH	SD
0	100.0	0.0
4	99.9	1.3
8	100.3	1.7
12	101.5	1.7
24	101.8	1.2
48	102.6	1.0
72	100.2	1.5
96	98.1	1.0

Supplementary data 8. Representative chromatogram of blank pooled HFH (t=0 min)

(a) Result of male fecal homogenate (b) Result of female fecal homogenate

(a)

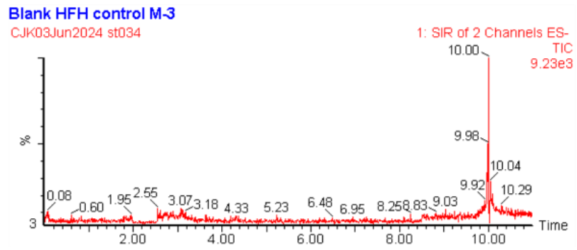

(b)

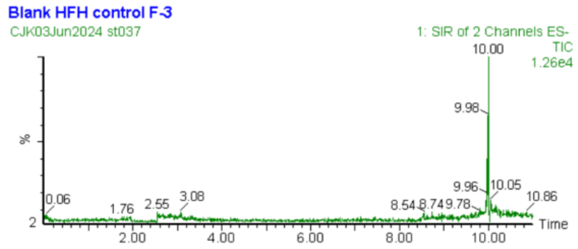

Supplement: Supplementary file 1 — Supplementary file1 (PDF 317 KB) [file 10068_2025_1959_MOESM1_ESM.pdf]
